# First retrospective study on pediatric nephrocalcinosis in Syria: clinical symptoms and causes

**DOI:** 10.3389/fped.2025.1604511

**Published:** 2025-07-28

**Authors:** Hadeel Ghanem, Mostafa Hassan, Sarena Swideah, Hala Wannous

**Affiliations:** ^1^ Department of Pediatric, Damascus University, Damascus, Syrian Arab Republic; ^2^ Department of Neurosurgery, Tartous University, Tartous, Syrian Arab Republic; ^3^ Department of Radiology, Tartous University, Tartous, Syrian Arab Republic; ^4^ Faculty Member of Pediatric Nephrology in Faculty of Medicine, Damascus University, Damascus, Syrian Arab Republic

**Keywords:** nephrocalcinosis, children, failure to thrive, renal disease, retrospective study, Middle East

## Abstract

**Background:**

Nephrocalcinosis is the deposition of calcium oxalate and phosphate in the kidneys. It is often asymptomatic and diagnosed via ultrasound. Symptoms may include hematuria or sterile leukocyturia. Based on echogenicity, it is classified as medullary or cortical. Although it may pose a problem in developing countries, it has not been adequately studied in the Middle East.

**Objectives:**

To investigate the clinical manifestations and outcomes of nephrocalcinosis for the first time in Syria. And to establish a primary database for such studies in Syria and the Middle East region.

**Methods:**

This retrospective study was conducted in a single pediatric nephrology department of a tertiary university hospital in Damascus, Syria. We collected the medical records of patients with a primary diagnosis of nephrocalcinosis between January 2014 and January 2018. All clinical, laboratory examination, treatment, and follow-up information were collected and analyzed.

**Results:**

Among 75 patients Forty-one (54%) were males and 34 (46%) were females, the median age at presentation was 18 months. The most presenting symptom was incidentally found in 39% of cases, then failure to thrive in 32% of cases, and recurrent urinary tract infections in 10.5% of cases. The most common leading cause of nephrocalcinosis was metabolic disorders in 68% followed by tubulopathy in 20%. The cause of nephrocalcinosis remained unknown in 8%, and renal malformations for 4% of the cases. During the study, five children developed end-stage renal disease (ESRD), and fifteen children died from different causes.

**Conclusion:**

This is the first Syrian study to review nephrocalcinosis cases. The most common leading cause of NC was metabolic disorders, most importantly, the patient with progressive nephrocalcinosis must be convinced that a high daily fluid intake is the most valuable therapeutic option.

## 1 Introduction

Nephrocalcinosis (NC) is defined as the deposition of both calcium oxalate and calcium phosphate in the kidney parenchyma and tubules. Kidney ultrasound (US) is a routine diagnostic procedure, nephrocalcinosis occurs either alone or in combination with calculi (1). NC is often asymptomatic, especially in early childhood, it is not unusual for nephrocalcinosis to be diagnosed during systematic renal ultrasound examination in high-risk infants or as part of the diagnostic evaluation of urinary tract infection. The first clinical symptoms, if any, are gross or microscopic hematuria and/or sterile leukocyturia, which may be misdiagnosed as urinary tract infection (1).

Nephrocalcinosis is classified according to the anatomic area involved, as medullary or cortical, and it is divided into three subtypes according to the degree of echogenicity (2), Common Causes of Nephrocalcinosis:

1. Hypercalcemia: is a common metabolic cause of Nephrocalcinosis, Idiopathic (primary) and secondary (3).
2. Hyperoxaluria: primary hyperoxaluria (PH) results from genetic mutations in different genes of glyoxylate metabolism (4) and secondary can result from a very high intake of oxalate in the diet and it is often associated with hypercalciuria (5).
3. Medications and Intoxications Causing Nephrocalcinosis: e.g., ceftriaxone, sulfonamides, ampicillin, amoxicillin, triamterene, acyclovir, guaifenesin, phenazopyridine, and oxypurinol Intoxication with ethylene glycol (6).
4. Other Disorders Complicated by Nephrocalcinosis Cystic Fibrosis and Other Malabsorption Syndromes (7).
5. Disorders with Abnormalities of Renal Tubule Function: A number of hereditary and acquired causes of renal tubule dysfunction result in nephrocalcinosis. Both primary and secondary forms of distal Renal tubular acidosis (d-RTA), such as those that occur with Wilson disease, Sjögren syndrome, type IA glycogen storage disease (8).

Early diagnosis is mandatory in a child with Nephrocalcinosis, because therapeutic measures may prevent kidney damage and even early renal failure, most importantly, the patient with progressive nephrocalcinosis must be convinced that a high daily fluid intake is the most valuable therapeutic option (9). When a reduction in renal function to a glomerular filtration rate of less than 30 ml/min/1.73 m^2^ occurs, renal replacement with transplantation or dialysis is needed promptly.

## 2 Materials and methods

### 2.1 Study population

A total of 75 patients diagnosed with nephrocalcinosis through ultrasonography at the Children University Hospital in Damascus were included in this study. Among these patients, 41 (54%) were males and 34 (46%) were females, all aged between one month and 14 years. The study period spanned from January 2014 to January 2018, with an average follow-up of one year. Our hospital is the only one to receive such clinical cases in the country. We reviewed patient files to gather essential information, including age, sex, medical history, family history, clinical presentations, as well as laboratory and imaging findings.

### 2.2 Definition

Diagnostic imaging for children includes ultrasonography and Computed Tomography (CT) scans. In this case, only 38 cases (50%) received CT scans due to financial constraints, and all of these children were diagnosed with nephrocalcinosis (100%). A study comparing ultrasonography and CT scanning for experimental nephrocalcinosis in rabbits found that ultrasonography had a higher sensitivity (96% compared to 64% for CT). Therefore, renal ultrasound is the preferred diagnostic imaging option for infants and children suspected of having kidney stones or nephrocalcinosis (10). During ultrasound examinations, multifocal scattered echogenic foci in the medulla, or a diffuse hyperechoic medulla, are identified as medullary nephrocalcinosis. This condition can be categorized into three subtypes based on the degree of echogenicity: (Grade I) Mild increase in the echogenicity around the border of the medullary pyramids, (Grade II) Mild diffuse increase in the echogenicity of the entire medullary pyramid, (Grade III) Greater, more homogeneous increase in the echogenicity of the whole of the medullary pyramid (11).

Focal bright hyperechogenic foci with posterior shadowing or gravity dependency at the collecting system were regarded as nephrolithiasis. We enrolled patients who had been diagnosed with nephrocalcinosis or medullary nephrocalcinosis based on sonographic readings provided by radiologists. We excluded patients with solitary nephrolithiasis without nephrocalcinosis, as well as neonates younger than one-month, premature infants diagnosed with nephrocalcinosis, and children older than 14 years who received treatment at another hospital.

The glomerular function was assessed by the estimated glomerular filtration rate (eGFR) calculated using the Schwartz formula, and levels of more than 90 ml/min/1.73 m^2^ were considered normal. For children <1 year of age, age-specific limits for serum creatinine were used to evaluate renal function (12).

### 2.3 Laboratory examinations

In all patients, the analysis of serum calcium, phosphorus, magnesium, uric acid, alkaline phosphatase, bicarbonate, and creatinine had been performed. Blood levels of parathyroid hormone and thyroid function tests were also conducted. Urine specimens were analyzed for both spot and 24 h excretion levels of calcium, creatinine, uric acid, and oxalate, as well as the calcium-to-creatinine ratio in randomized urine samples.

### 2.4 Molecular analysis

Genetic diagnosis was performed by screening DNA for mutations in the AGXT (alanine-glyoxylate aminotransferase) gene in 23 patients. The underlying cause of nephrocalcinosis was determined based on calcium excretion levels, which are considered normal if they are below 4 mg (0.1 mmol) per kg of body weight per day. Patients with primary hyperoxaluria underwent molecular analysis.

### 2.5 Treatment

Treatment options varied from observation to medical intervention and surgery. Patients were classified into three groups:

1. The first group includes unknown cases that were observed and routinely followed up, which involves ultrasonography, urine examination, and renal function tests.
2. The second group consists of metabolic abnormality and tubulopathy which had medical treatments such as hydration, urine alkalization using potassium citrate, hydrochlorothiazide for idiopathic hypercalciuria (IH) and renal tubular acidosis, and pyridoxine for patients with primary hyperoxaluria (PH1), and surgical treatment for hyperparathyroidism (only one case), and dialysis for End-stage renal disease (ESRD) cases.
3. The third group involves renal malformations which had antibiotic treatment and routine follow-up.

A large daily fluid intake of more than 2 liters per 1.73 m^2^ is an effective therapeutic option unless the patient is experiencing oliguria (Table 1).

**Table 1:** Treatment of nephrocalcinosis; values represent numbers (percentage).

Cause of nephrocalcinosis		Treatment	No. (%)
Group with unknown causes		Follow-up Wait and see	6 (8%)
Group with metabolic abnormality or tubulopathy	Idiopathic hypercalciuria (IH)	Hydrochlorothiazide AND Potassium citrates	20 (26.5%)
		Dialysis for ESRD	1 (1.5%)
	Primary Hyperoxaluria type 1(PH1)	Pyridoxine	12 (16%)
		Dialysis for ESRD	2 (2.5%)
	Hyperoxaluria for other causes*	Low oxalate diet	13 (17%)
	Renal tubular acidosis	Potassium and sodium citrates	8 (10.5%)
		Dialysis for ESRD	2 (2.5%)
	Bartter syndrome	Indomethacin and potassium-sparing diuretics	3 (4%)
	Dent Disease	Hydrochlorothiazide	1 (1.5%)
	Acquired tubular nephropathy (sickle cell disease)	Potassium and sodium citrates	1 (1.5%)
	Hyperparathyroidism	Surgical treatment	1 (1.5%)
		Medical treatment	2 (2.5%)
Group with renal malformations		Preventive antibiotic Wait and see	3 (4%)
		Surgical treatment	0(0%)

* Other causes include Primary Hyperoxaluria type 2 and secondary Hyperoxaluria.

### 2.6 Statistical analysis

We used the Microsoft Excel program [version 2016] to collect the data. The collected data was analyzed by SPSS [version 25] for Mac using the Student's *T*-test, the Mann–Whitney *U*-test, chi-square test, Fisher's exact test, and logistic regression analysis, with *P* values of <0.05 considered to be significant. Clinical trial number: not applicable.

## 3 Results

### 3.1 Demographic

The prevalence of nephrocalcinosis is 0.51%. A positive family history of NC was noted in 22 patients, accounting for 29%. The patients were classified by age into three groups: 1 month to 1 year, over 1 year to under 5 years, and over 5 years to under 13 years. The prevalence of NC in these age groups was 72%, 14.5%, and 13.5%, respectively. The median age at presentation was 18 months.

### 3.2 Presenting symptoms at diagnosis

The most common presenting symptom was an incidental finding, observed in 39% of cases, followed by failure to thrive in 32%, recurrent urinary tract infections in 10.5%, polyuria and polydipsia in 8%, abdominal pain in 5.5%, hematuria in 2.5%, and convulsions secondary to hypocalcemia in 2.5% of cases.

At the time of diagnosis, 17 patients (22.5%) had decreased renal function, and 42 patients (56%) had a urinary infection. Based on the degree of echogenicity observed in ultrasounds, cortical nephrocalcinosis was noted in 4 patients (5.5%), three of whom had primary hyperoxaluria, and one had sickle cell disease. In contrast, medullary nephrocalcinosis was found in 71 patients (94.5%). Medullary nephrocalcinosis was further classified into three subtypes according to the degree of echogenicity (Table 2).

**Table 2:** Grading scale for medullary nephrocalcinosis.

Grading	No (%)
Grade I	24 (33.5%)
Grade II	32 (45%)
Grade III	15 (21.5%)

Hypercalciuria was identified in 53 patients (70.5%). Urolithiasis and nephrocalcinosis were found in 23 patients (30.5%), with urolithiasis primarily affecting the upper urinary tract.

### 3.3 Etiology

The most common leading cause of nephrocalcinosis was metabolic disorders, accounting for 68% of cases, followed by tubulopathy at 20%. In 8% of cases, the cause of nephrocalcinosis remained unknown, while renal malformations were present in only 4% (Table 3).

**Table 3:** Causes of nephrocalcinosis; values represent numbers(percentage).

Cause of NC		No. (%)
Idiopathic hypercalciuria (IH)		21 (28%)
Hyperparathyroidism		3 (4%)
Hyperoxaluria	Primary Hyperoxaluria type 1(PH1)	14 (18.5%)
	Hyperoxaluria for other causes	13 (17%)
Hereditary tubular nephropathy	dRTA type 1	7 (9%)
	pRTA type 2	2 (2.5%)
	RTA type 4	1 (1.5%)
	Bartter syndrome	3 (4%)
	Dent Disease	1 (1.5%)
Acquired tubular nephropathy (sickle cell disease)		1 (1.5%)
Renal malformations		3 (4%)
Unknown		6(8%)

dRTA, distal renal tubular acidosis; pRTA, proximal renal tubular acidosis.

Urinary oxalate excretion was elevated, with more than 0.46 mmol/1.73 m^2^/24 h identified in 27 patients. Among these, 23 patients exhibited hyperoxaluria greater than 1.0 mmol/1.73 m^2^/24 h, and they were screened for mutations in the AGXT gene. Fourteen of these patients were diagnosed with Primary Hyperoxaluria type 1 and tested positive for the mutation.

### 3.4 Outcome and follow-up

Throughout the study, five patients developed end-stage renal disease, which accounted for (6.7%) of the participants. Among them, two had primary hyperoxaluria type 1, one had proximal renal tubular acidosis (pRTA) type 2, one had renal tubular acidosis (RTA) type 4, and one had idiopathic hypercalciuria. Three of the patients with ESRD underwent hemodialysis three times a week, while two received peritoneal dialysis.

Additionally, 15 patients died, representing (20%). Twelve of these patients had chronic kidney disease (CKD) stages 4–5 and passed away due to complications related to their condition. One patient died following surgery for hyperparathyroidism, and two patients experienced cardiac arrests after arrhythmias caused by electrolyte imbalances. Unfortunately, follow-up information was missed for four patients (5.3%) due to wartime circumstances (Table 4).

**Table 4:** Represent growth parameter, laboratory results at presentation and after follow-up.

Parameter	Presentation	Follow-up	*P (value)*
Age (months)	18 (1–156)	24 (14–170)	
z-score	−3.5 (−5.5 to +1)	−2.9 (−3 to +1)	0.02
eGFR (ml/min/1.73 m^2^)	5 (25 to 110)	78 (10 to 110)	0.001

eGFR, estimated glomerular filtration rate.

## 4 Discussion

Nephrocalcinosis in children is a significant concern, and we currently have limited information on the topic (13). This study is the first of its kind in our country, and our center is the only referral facility for such cases of nephrocalcinosis. Therefore, the findings of this study might reflect the epidemiology of NC in our country. Pediatric nephrologists continuously monitor patients with nephrocalcinosis. Any sign or symptom of nephrocalcinosis should prompt further investigation. Patients diagnosed with nephrocalcinosis require regular follow-up through laboratory tests and imaging studies. NC can affect all ages, although it primarily appears to manifest in the first years of life (14, 15).

In the study conducted by Sofia B. et al. (14), which involved 35 patients aged from one day to 17 years between 2008 and 2017, the most common presenting symptom was failure to thrive, observed in 34% of the cases. Additionally, nephrocalcinosis was diagnosed incidentally in 23% of the patients. Similarly, in the study by Ammenti A. et al. (15), which included 41 children, failure to thrive during the first year of life was noted in 41% of cases. Nephrocalcinosis was detected incidentally in 24% of those patients. In contrast to these findings, our study revealed that nephrocalcinosis is primarily asymptomatic, with incidental detection being the most common presenting symptom. Thus, we highly recommend that all children under one year receive an abdominal ultrasound.

In the studies conducted by Mantan M et al. (16) and Jwaher T Al-Bderat et al. (17), the etiology of NC included tubulopathies such as distal renal tubular acidosis, as well as idiopathic hypercalciuria and hyperoxaluria, along with a number of unknown cases. Ramya K et al. (18) also reported that distal RTA, primary hyperoxaluria, and Bartter syndrome were common etiologies of nephrocalcinosis. Our study found that metabolic causes were the most prevalent, largely due to consanguineous marriages in our country. In our cohort, 22 patients (29%) had a positive family history of nephrocalcinosis, whereas previous studies (14, 17) reported this at 40%.

In our study, five patients (6.5%) developed end-stage renal disease. In the study by Doğan et al. (19), which included 27 patients, chronic renal insufficiency occurred in 5 patients (11.5%). While Jwaher T. Al-Bderat et al. (17) reported that eight patients (12.5%) had ESRD. Although our study included a larger sample size than the previous two studies, we observed a lower incidence of kidney failure.

## 5 Conclusion

Predisposing factors for nephrocalcinosis (NC) are known in patients and should be thoroughly investigated. Early diagnosis is crucial to prevent serious outcomes that could lead to end-stage renal disease (ESRD). Ultrasonography is the most valuable imaging method. As this study is the first of its kind regarding nephrocalcinosis in our country, it serves as a foundation for further research on this topic. This study not only lays the groundwork for a prospective study but also creates an important database to enhance our clinical understanding of nephrocalcinosis manifestation in Syria and maybe surrounding areas.

## Data Availability

The raw data supporting the conclusions of this article will be made available by the authors, without undue reservation.
